# Autosomal recessive cerebellar ataxias

**DOI:** 10.1186/1750-1172-1-47

**Published:** 2006-11-17

**Authors:** Francesc Palau, Carmen Espinós

**Affiliations:** 1 Genetics and Molecular Medicine Unit, Instituto de Biomedicina, CSIC, Jaume Roig, 11 46010 Valencia, Spain; 2 Centre for Biomedical Research on Rare Diseases (CIBERER), Instituto de Salud Carlos III, Valencia, Spain

## Abstract

Autosomal recessive cerebellar ataxias (ARCA) are a heterogeneous group of rare neurological disorders involving both central and peripheral nervous system, and in some case other systems and organs, and characterized by degeneration or abnormal development of cerebellum and spinal cord, autosomal recessive inheritance and, in most cases, early onset occurring before the age of 20 years. This group encompasses a large number of rare diseases, the most frequent in Caucasian population being Friedreich ataxia (estimated prevalence 2–4/100,000), ataxia-telangiectasia (1–2.5/100,000) and early onset cerebellar ataxia with retained tendon reflexes (1/100,000). Other forms ARCA are much less common. Based on clinicogenetic criteria, five main types ARCA can be distinguished: congenital ataxias (developmental disorder), ataxias associated with metabolic disorders, ataxias with a DNA repair defect, degenerative ataxias, and ataxia associated with other features. These diseases are due to mutations in specific genes, some of which have been identified, such as frataxin in Friedreich ataxia, **α**-tocopherol transfer protein in ataxia with vitamin E deficiency (AVED), aprataxin in ataxia with oculomotor apraxia (AOA1), and senataxin in ataxia with oculomotor apraxia (AOA2). Clinical diagnosis is confirmed by ancillary tests such as neuroimaging (magnetic resonance imaging, scanning), electrophysiological examination, and mutation analysis when the causative gene is identified. Correct clinical and genetic diagnosis is important for appropriate genetic counseling and prognosis and, in some instances, pharmacological treatment. Due to autosomal recessive inheritance, previous familial history of affected individuals is unlikely. For most ARCA there is no specific drug treatment except for coenzyme Q10 deficiency and abetalipoproteinemia.

## Disease name and synonyms

Autosomal recessive cerebellar ataxias (ARCA)

Early onset cerebellar ataxias (EOCA)

Autosomal recessive spinocerebellar ataxias

## Definition and classification

Autosomal recessive cerebellar ataxias (ARCA) belong to the wider group disorders known as inherited ataxias [1-3]. ARCA are neurological disorders characterized by degeneration or abnormal development of cerebellum and spinal cord, autosomal recessive inheritance and, in most cases, early onset occurring before the age of 20. This group encompasses a large number of rare diseases, the most frequent being Friedreich ataxia.

Different criteria have been used to classify ARCA and issues of classification still remain controversial. In the 80s of the last century, Harding proposed a clinical classification for inherited ataxias based on two criteria: the age of onset and pathological mechanisms [4,5]. Most of early onset ataxias (before age of 20 years) show autosomal recessive inheritance and may be classified as ARCA. Some of them are not progressive disorders and are associated with impaired development of the cerebellum and its connections; they were considered congenital ataxias. Other have a metabolic cause and show either progressive or intermittent natural history. The most frequent ataxias in Harding classification were degenerative ataxias with unknown cause. However, in the last ten years molecular genetic studies have changed the global panorama of inherited ataxias. Koenig has used topographical and pathophysiological criteria for ARCA; he distinguished sensory and spinocerebellar ataxias, cerebellar ataxias with sensory-motor peripheral neuropathy and pure cerebellar ataxia [6]. Recently, the group of Filla suggested a pathogenic approach to classify hereditary ataxias [7]. These authors did not consider any genetic, pathological or natural history-based criteria, and divided the disorders in mitochondrial ataxias (including Friedreich ataxia), metabolic ataxias, ataxias associated with defective DNA repair, ataxias with abnormal protein folding and degradation, ataxias caused by channelopathies, and a miscellaneous group with unknown pathogenic mechanisms.

In order to offer a clinically-oriented classification of ARCA, we use here the classical criteria that take into account both genetic inheritance and natural history of the clinical symptoms. The Mendelian criterion allows to consider ARCA as a group of inherited ataxias (including most of the congenital non-progressive ataxias), which differ either from the group of autosomal dominant cerebellar ataxias (late onset spinocerebellar ataxias and episodic ataxias) or the small group of X-linked ataxias. Consequently, we propose a classification of ARCA as follows: 1) congenital or developmental ataxias; 2) metabolic ataxias, including ataxias caused by enzymatic defects; 3) ataxia due to DNA repair defects; 4) degenerative and progressive ataxic disorders that include ataxias with known cause and pathogenesis (such as Friedreich ataxia), and ataxias of unknown etiology; 5) ARCA with additional features (Table 1). It should be pointed out that this classification is conditioned by the current and rapidly evolving knowledge on the molecular mechanisms of ataxia. This is especially important for the development of new treatments based on the knowledge of the pathogenesis of disease.

**Table 1:** Genetic data on ARCA Disorders

	**Protein (*GENE *or LOCUS)**	**Location**	**MIM**
***Congenital ataxias***			

Joubert syndrome			
JBTS1 (cerebelloparenchymal disorder IV, CPD IV)	(JBTS1)	9q34	#213300
JBTS2 (CORS2)	(JBTS2)	11p12-p13.3	#608091
JBTS3	AHI1 (*AHI1*)	6q23	#608629
JBTS4 (nephronophthisis 1)	(*NPHP1*)	2q13	#609583
JBTS5	nephrocystin-6 (*CEP290 or NPHP6*)	12q21.32	#610188
Cayman ataxia	Cayataxin, (*ATCAY*)	19p13.3	#601238

***Metabolic ataxias***			

Ataxia with isolated vitamin E deficiency (AVED)	Alpha-tocopherol transfer protein (α-*TTP*)	8q13	#277460
Abetalipoproteinemia	Microsomal trygliceride transfer protein (*MTP*)	4q22-q24	#200100
Cerebrotendinous xanthomatosis	Sterol 27-hydroxylase (*CYP27*)	2q33-qter	#213700
Refsum disease	Phytanoyl-CoA hydrixylase (*PhyH*) Peroxisomal biogenesis factor-7 (*PEX7*)	10pter-p11.2 6q22-q24	#266500

***DNA repair defects***			

Ataxia telangiectasia	*ATM*	11q22.3	#208900
Ataxia with oculomotor apraxia 1 (AOA1)	Aprataxin (*APTX*)	9p13	#208920
Ataxia with oculomotor apraxia 2 (AOA2) or SCAR1	Senataxin (*SETX*)	9q34	#606002
Ataxia-telangiectasia-like disorder (ATLD)	*MRE11A*	11q21	#604391
Spinocerebellar ataxia with axonal neuropathy (SCAN1)	Tyrosyl-DNA phosphodiesterase 1 (*TDP1*)	14q31	#607250
Xeroderma Pigmentosum (XP)			
XP of complementation group A	XPA (*XPA*)	9q22.3	#278700
XP of complementation group B	XPB/ERCC3·(*XPB/ERCC3*)	2q21	#133510
XP of complementation group C	XPC (*XPC*)	3p25	#278720
XP of complementation group D	XPD/ERCC2 (*XPD/ERCC2*)	19q13.2-q13.3	#278730
XP of complementation group E	XPE (*DDB2*)	11p12-p11	#278740
XP of complementation group F	XPF/ERCC4 (*XPF/ERCC4*)	16p13.3-p13.3	#278760
XP of complementation group G	XPG/ERCC5 (*XPG/ERCC5*)	13q32-q33	#133530
XP variant (XPV) or XP with normal DNA repair rates	POLH (*POLH*)	6p21.1-p12	#278750

***Degenerative ataxias***			

Friedreich ataxia	Frataxin (*FRDA or FXN*)	9q13	#229300
Mitochondrial recessive ataxic syndrome (MIRAS)	Polymerase *γ *(*POLG*)		*174763
Charlevoix-Saguenay spastic ataxia	Sacsin (*SACS*)	13q12	#270550
Early onset cerebellar ataxia with retained tendon reflexes (EOCARR)		13q11-12	#212895
Infantile onset spinocerebellar ataxia (IOSCA)	Twinkle (*C10orf2*)	10q22.3-q24.1	#271245
Marinesco-Sjögren syndrome:			#248800
Classical MSS	SIL1 (*SIL1*)	5q32	
MSS with myoglobinuria		18qter	
Coenzyme Q_10 _deficiency with cerebellar ataxia	?		#607426
Posterior column ataxia and retinitis pigmentosa (PCARP)	(AXPC1)	1q31	#609033

## Etiology

Autosomal recessive cerebellar ataxias are Mendelian inherited disorders. Every disease belonging to this group is caused by mutations in a specific gene. Some of ARCA show genetic heterogeneity due to mutation(s) in more that one gene/locus (Table 1).

## Clinical description

### 1. Congenital (developmental) ataxias

Some rare developmental anomalies, such as dysgenesis or agenesis of the vermis, cerebellar hemispheres or parts of the brainstem, may give rise to congenital ataxias, and most of them are associated with an autosomal recessive inheritance. In the last years, some congenital ataxias such as Joubert syndrome have also been associated with specific chromosomal loci and genes.

#### Joubert syndrome

Joubert syndrome (JBTS) is a rare autosomal recessive brain disorder characterized by i) absence of cerebellar vermis and ii) presense of "molar tooth sign" (MTS). MTS is formed by abnormal configuration of the superior cerebellar peduncles (SCPs) that connect the cerebellum to the midbrain and thalamus. The most common clinical manifestations include infantile onset of cerebellar ataxia, nystagmus, vertical gaze paresis, ptosis, retinopathy, mental retardation, and episodic hypernea or apnea of the newborn. There is agenesis of cerebellar vermis. Most of the approximately 200 patients reported to date have additional clinical features associated with those of the classical JBTS. The related clinical features define a large spectrum of syndromes with MTS (such as Senior-Löken syndrome), which (together with JBTS) are termed Joubert syndrome related disorders (JSRD) or MTS related syndromes [8,9]. To date, five loci associated with JBTS have been mapped to chromosomes 9q34.3 (JBTS1), 11p11.2-q12.3 (JBTS2), 6q23 (JBTS3), 2q13 (JBTS4), and 12q21 (JBTS5) [10-13]. Mutations in the *AHI1 *gene have been reported in three JBTS3-linked families presenting with a pure cerebellar phenotype [14]. Moreover, the *NPHP1 *gene deletion associated with juvenile nephonophthisis has been identified in subjects affected by a mild form of JBTS [15,16]. Recently, mutations in the *CEP290 *gene have been found both in patients with JBTS5 [17] and nephronopthisis [18]. *CEP290 *or *NPHP6 *encodes for nephrocystin-6, a centrosomal protein that modulates the activity of ATF4, a transcription factor implicated in the cAMP-dependent renal cyst formation.

#### Cayman cerebellar ataxia

Cayman cerebellar ataxia is another congenital ataxia for which the causative gene has been identified. Individuals with Cayman ataxia have hypotonia from birth, psychomotor delay and non-progressive cerebellar dysfunction, including truncal and limb ataxia, dysarthria, nystagmus, and intention tremor. Imaging studies show cerebellar hypoplasia. The disease has been observed in an isolated population of the Grand Cayman Island; it is caused by mutations in the *ATCAY *gene [19,20]. The encoded protein, cayataxin, has a CRAL-TRIO domain. This motif is also found in the **α**-tocopherol transfer protein that causes ataxia with vitamin E deficiency (AVED).

### 2. Metabolic ataxias

Metabolic ataxias include:

• progressive ataxias;

• disorders associated with intermittent ataxia (*e.g. *syndromes with hyperammonemias, aminoacidurias, and disorders of pyruvate and lactate metabolism);

• metabolic disorders in which ataxia occurs as a minor feature (*e.g. *metachromatic leukodystrophy, adrenoleukodystrophy, and sphingomyelin storage disorders).

Here we just mention some of the most relevant progressive ataxia involving metabolism.

#### Ataxia with isolated vitamin E deficiency

Ataxia with isolated vitamin E deficiency (AVED) [21] is a hereditary ataxia caused by mutations in the **α**-tocopherol transfer protein gene, ***α***-*TTP *[22,23]. AVED has been described in primary metabolic defects such as abetalipoproteinemia, or secondary to fat malabsortion, chronic cholestasis, pancreatic insufficiency, or cystic fibrosis. In AVED, the unique biochemical abnormality is the very low plasma level of vitamin E, and respectively, the low level of the unique form of vitamin E present in the mammalian serum, RRR-**α**-tocopherol.

**α**-TTP is the protein responsible for the specific transfer of vitamin E to the nascent very low-density lipoproteins (VLDL). Patients with AVED have normal vitamin E absorbtion in the intestine, but poor conservation of plasma RRR-**α**-tocopherol due to impaired secretion of RRR-**α**-tocopherol in VLDL; thus VLDL represent the primary defect in the pathogenesis of the disease.

AVED is characterized by progressive sensory and cerebellar ataxia usually beginning before age of 20 years (range 2–52 years). Patients with AVED show clinical signs similar to those in Friedreich ataxia, including gait and limb ataxia, dysarthria, lower limb areflexia, loss of vibration and positional sense, and bilateral Babinski sign. In contrast, cardiomyopathy and glucose intolerance are much less frequent, and head titubation and dystonia are observed in some patients only. Diagnosis is based on the finding of low serum vitamin E values (< 2.5 mg/L; normal values 6–15 mg/L) in absence of malabsortion. In contrast to abetalipoproteinemia, AVED patients have a normal lipidogram and normal red blood cell morphology with no acantocytes. Electrophysiological studies show signs of axonal sensory neuropathy with moderate reduction of sensory nerve action potentials (SNAP) and normal motor nerve conduction velocities. Electromyogram is either normal or neurogenic, with polyphasic recordings [24,25].

AVED is particularly frequent in countries from the Mediterranean basin and most of cases come from North African populations. The 744delA frameshift mutation is the most frequently found defect and is distributed as a result of a founder effect [23]. The most frequent mutation identified in the Japanese population is an amino acid substitution, H101Q, associated with a mild phenotype [26,27].

Treatment is based on vitamin E supplements. Administration to adults of 800 mg RRR-**α**-tocopherol twice daily leads to an increased plasma levels of **α**-tocopherol and reduction of symptoms and signs.

#### Abetalipoproteinemia

Abetalipoproteinemia or Bassen-Kornzweig syndrome is an autosomal recessive inherited inborn error of lipoprotein metabolism caused by molecular abnormalities in the microsomal trygliceride transfer protein (MTP), whose gene maps to chromosome 4q22-q24 [28-31]. The MTP catalyzes the transport of triglyceride, cholesteryl ester and phospholipid between phospholipid surfaces. Thus, the assembly or secretion of plasma lipoproteins that contain apolipoprotein B is thought to be the basic pathogenetic defect.

Clinical features include malabsorption syndrome, pigmentary degeneration of the retina and progressive ataxic neuropathy. Red cells show a peculiar "thorny" deformation called acanthocytosis. The neurological symptoms are directly related to the deficiency of liposoluble vitamin E. Total cholesterol is low (<70 mg/dL) and triglycerides are almost undetectable. Lipoprotein profile is characterized by absent low-density lipoproteins (LDL) and VLDL. Symptoms observed in some patients with abetalipoproteinemia are indistinguishable from those of patients with homozygous hypobetalipoproteinemia (HBLP), a codominant genetic disorder characterized by decreased or absent plasma levels of apolipoprotein (apo) B, due to mutations in the *apo B *gene (chromosome 2p24) [32,33] (for review see [34]).

#### Cerebrotendinous xanthomatosis

Cerebrotendinous xanthomatosis (CTX) or sterol 27-hydroxylase deficiency is an autosomal recessive disorder characterized by defect in bile acid biosynthesis and storage of sterols, and early childhood onset. It has been reported in approximately 200 people worldwide, although the prevalence seems higher in Sephardic Jewish of Moroccan origin [35]. Clinical characteristics include xanthomas of the Achilles and other tendons, juvenile cataracts, early atherosclerosis and progressive neurological disorder with cerebellar ataxia beginning after puberty, as well as systemic spinal cord involvement and dementia. Intelligence is low to normal. Large deposits of cholesterol and cholestanol are found in virtually all tissues, particularly in Achilles tendon, brain and lungs. CTX shares some clinical manifestations (including xanthomas and coronary atherosclerosis) with other lipid storage disorders such as familial hypercholesterolemia and phytosterolemia. However, progressive neurologic symptoms, cataracts and mild pulmonary insufficiency distinguish CTX from these two disorders [36].

The disease is caused by mutations in the sterol 27-hydroxylase gene (*CYP27*) mapped to chromosome 2q33-qter [37]. Approximately 40 different mutations of the *CYP27 *gene have been identified in CTX patients from various populations. Most of mutations are related to the adrenodoxin-binding site and the heme-binding site, although the location of some mutations suggests other putative binding sites to other proteins [38]. The *CYP27 *gene encodes for a mitochondrial cytochrome P-450, which hydroxylates a variety of sterols at C27 position, in association with two protein cofactors, adrenodoxin and adrenodoxin reductase. In the bile acid synthesis pathway, sterol 27-hydroxylase catalyzes the first step in the oxidation of the side chain of sterol intermediates. In CTX, cholestanol (5-alpha-dihydro derivative of cholesterol) has elevated concentrations in all tissues (compared to cholesterol). Diagnosis is made by demonstrating abnormal values of cholestanol in serum and tendons. Plasma cholesterol may be low or normal.

Treatment with cholic acid or chenodeoxycholic acid is indicated. Other treatments include the use of pravastin, a 3-hydroxy-3-methylglutaryl (HMG)-CoA reductase inhibitor, or the combination of both chenodeoxycholic acid and pravastin (for review see [39]).

#### Refsum disease

Refsum disease (also named phytanic acid oxidase deficiency, heredopathia atactica polyneuritiformis or hereditary motor and sensory neuropathy IV (HMSN IV), is a rare inherited disorder characterized by defective peroxisomal alpha oxidation of the fatty acids. This defect impairs the metabolism of branched chain fatty acids like phytanic acid (Phyt) and, as a consequence, Phyt accumulates in the blood and other tissues.

The age of onset varies from early childhood to 50 years of age, but most patients have clear-cut manifestations before age of 20 years. The three main clinical features are retinitis pigmentosa, chronic polyneuropathy, and cerebellar ataxia. Most of cases have anosmia, deafness, cardiac arrhythmias, bony and skin abnormalities.

Other peroxisome biogenesis disorders associated with high levels of Phyt are Zellweger syndrome, neonatal adrenoleukodystrophy, infantile Refsum disease, and rhizomelic chondrodysplasia punctata. The first three of them represent a continuum of overlapping features, the most severe being the Zellweger syndrome, and the less severe infantile Refsum disease. Rhizomelic chondrodysplasia punctata is characterized by a distinct phenotype.

Refsum disease is an autosomal recessive trait that is genetically heterogenous. The *PHYH *(or *PAHX*) gene placed on chromosome 10pter-p11.2 is responsible for the disorder in most of patients [40]. The *PHYH *gene spans 21 Kb, contains nine exons and encodes for the phytanoyl-CoA hydroxylase (PhyH) that catalyses the first step in the alpha oxidation of Phyt. Thus, in the majority of patients with Refsum disease, PhyH activity is deficient [41,42]. Different types of mutations have been found distributed along the *PHYH *gene [43]. Recently, Kahlert *et al. *[44] have suggested that the cytotoxic effect of Phyt seems to be related to a combination of effects on Ca^2+ ^regulation, mitochondrial depolarization and increased reactive oxygen species (ROS) generation in brain cells.

A second locus involved in Refsum disease is the *PEX7 *gene (chromosome 6q21-q22.2) that consists of 10 exons (102 Kb) [45,46]. To date, only three Refsum patients have been reported with mutations in this gene; mutations in the *PEX7 *gene have been found causative for other disorders, such as rhizomelic chondrodysplasia punctata [47]. *PEX *genes encode for peroxisomal assembly proteins called peroxins, which are required for the import of matrix proteins into peroxisomes.

### 3. DNA repair defects

#### Ataxia-telangiectasia

Ataxia-telangiectasia (A-T) is a multisystem disease characterized by progressive cerebellar ataxia, oculomotor apraxia, oculocutaneous teleangiectasia, coreoathetosis, recurrent sinopulmonary infections, variable immunodeficiency state with involvement of cellular and humoral immunity, high risk of malignancy (especially leukemia and lymphoma), and enhanced sensitivity to ionizing radioactivity. It is the more common autosomal recessive ataxia after Friedreich ataxia, with an estimated prevalence of 1–2.5/100,000. In most of the cases, the disease begins at age of 2 to 4 years, the first symptom being cerebellar ataxia. Oculomotor signs are present is almost all patients. Teleangiectasias are the second hallmark of the disorder and appear between age of 2 and 8 years. Cerebellar degeneration is progressive until adulthood and patients need a wheelchair by the age of 10. Lifespan is reduced but quality of life has improved and patients survive more than 20 years of age (some of them survive into their 40s and 50s) (for review see [48]). Neuropathologic studies show an atrophic cerebellum, predominantly throughout the vermis and less in the lobules. There is reduced number and abnormal arborisation of Purkinje cells, and marked thinning of the molecular layer and granular layer.

Classical complementation experiments on fibroblasts culture from patients suggested that there were a number of genes implicated in the diseases pathogenesis. However, linkage studies showed only one locus on chromosome 11q23 accounting for the disease in different populations [49,50]. Mutation analysis of the *ATM *gene has confirmed the genetic homogeneity of A-T. The gene has 66 exons spanning more than 150 kb. A transcript of 12 kb encodes a protein of 3056 amino acids with 370 kDa, which is a member of the phosphoinositol-3 kinase (PI-3K)-like serine/threonine kinases involved in the cell cycle checkpoint control and DNA repair [51,52]. More than 200 distinct mutations have been reported associated with A-T. Mutations are distributed throughout the entire gene and involve almost all coding exons [53]. Most of patients are compound heterozygous for mutations that give truncated proteins, although missense mutations can alter the protein function [54]. Some A-T cases have atypical clinical manifestations with a milder phenotype that present one or more of the following symptoms: later onset of the first symptoms, slower progression, longer lifespan, and lower levels of chromosomal instability and cellular radiosensitivity [55]. These patients are usually compound heterozygous for a more severe mutation with a milder mutation that makes possible the expression of a small amount of protein [56,57]. As the majority of patients carry unique mutations, mutation analysis is possible for the clinical diagnosis of A-T. When the mutation is unknown or sequence analysis is not available, prenatal diagnosis is based on linkage analysis using flanking linked markers.

Diagnosis is suspected in young children who show signs of cerebellar ataxia, oculomotor apraxia and telangiectasias of the conjunctivae. Magnetic resonance imaging (MRI) examination shows cerebellum atrophy. Other tests that may support the diagnosis are: serum alpha-fetoprotein is elevated above 10 ng/ml in more than 90% of patients; cytogenetic analysis shows a 7:14 translocation (t [7;14] [q11;q32]) in 5–15% of affected individuals; colony *in vitro *assay (irradiation of colony formation of lymphoblastoid cells) is a very sensitive test but takes approximately three months and is available only in specialized centres. Mutation analysis (available in specialized laboratories only) deserves a special attention as it is not only useful to confirm the clinical diagnosis but is also useful for carrier detection, genetic counseling and prenatal diagnosis.

#### Ataxia-telangiectasia-like disorder

Ataxia telangectasia-like disorder (ATLD) is a very rare syndrome that shares similarities with A-T and Nijmegen breakage syndrome. Patients' cells show chromosomal instability, increased sensitivity to ionizing radiation, defective induction of stress-activated signal transduction pathways, and radioresistant DNA synthesis [58,59]. The causative gene, *MRE11A*, maps very close to *ATM *(chromosome 11q), thus only a very detailed linkage analysis would separate ATLD from A-T purely on the basis of genetic data [57].

#### Ataxia with oculomotor apraxia 1

Ataxia with oculomotor apraxia 1 (AOA1) is characterized by early onset gait ataxia (between 2 and 6 years of age), dysarthria, limb dysmetria (later in disease course), oculomotor apraxia, distal and symmetric muscle weakness and wasting, mild loss of vibration and joint position sense, and slow progression. Some patients show dystonia, masked facies or mental retardation. Laboratory studies show a motor and sensory axonal neuropathy, mild loss of large myelinated axons, cerebellar and brainstem atrophy on MRI, hypoalbuminemia and hypercholesterolemia [60,61]. The disease has been originally reported in Portuguese [61] and Japanese [62] patients. In Japan, AOA1 seems to be the most frequent recessive ataxia, whereas in Portugal it is the second one, after Friedreich ataxia [63].

The causative *APTX *gene, located on chromosome 9p13.3, has seven exons and encodes a novel protein, aprataxin [64]. Alternative splicing in exon 3 generates two distinct isoforms, the longer transcript encodes for a 342 amino acid protein, while the shorter one encodes a 174 amino acid protein. Aprataxin is a nuclear protein composed of three domains that share homology with the amino-terminal domain of polynucleotide kinase 3'-phosphatase (PNKP), histidine-triad (HIT) proteins and DNA-binding C2H2 zinc-finger proteins, respectively. PNKP is involved in DNA single-strand break repair (SSBR) following exposure to ionizing radiation and ROS [65]. Recently, involvement of aprataxin in the DNA sSSBR-machinery has been demonstrated [66]. Thus, AOA1 may be classified within the group of ataxias associated with DNA repair defects. Due to its capacity to interact with proteins involved in DNA repair, aprataxin could influence the cellular response to genotoxic stress [67].

#### Ataxia with oculomotor apraxia 2

Ataxia with oculomotor apraxia type 2 (AOA2), also referred as non-Friedreich spinocerebellar ataxia type 1 (SCAR1), is an autosomal recessive disorder that represents approximately 8% of non-Friedreich ARCA [68]. It is characterized by spinocerebellar ataxia with onset between 11 and 22 years, choreoathetosis, dystonic posturing with walking, and is occasionally associated with oculomotor apraxia, and elevated values of gamma-globulin, alpha-protein and creatin kinase (CK) [68]. Cerebellar atrophy is observed in some patients. Electrophysiology studies show absence of sensory potentials.

The causative gene has been mapped to chromosome 9q34. The gene has recently been isolated and characterized [69]. It encodes senataxin (SETX), a 2,677-amino acid protein that contains at its C-terminus a classic 7-motif domain found in the superfamily 1 of helicases. Senataxin may act in the DNA repair pathway and also may be a nuclear RNA helicase with a role in the splicing machinery [69]. No evidence of chromosome instability or sensitivity to ionizing radiation has been observed in cells from affected individuals. Mutations in *SETX *gene have been identified also in the autosomal dominant form of amyotrophic lateral sclerosis, known as ALS4 [70].

#### Spinocerebellar ataxia with axonal neuropathy

Spinocerebellar ataxia with axonal neuropathy (SCAN1) is a disorder characterized by recessive ataxia with peripheral axonal motor and sensory neuropathy, distal muscular atrophy, and pes cavus and stepagge gait, as described in Charcot-Marie-Tooth disease. Genetic studies in a large Saudi Arabian family mapped the disease to chromosome 14q31 and identified a homozygous mutation in the gene encoding topoisomerase I-dependent DNA damage repair enzyme (*TDP1*) [71]. Patients had history of seizures, mild brain atrophy, mild hypercholesterolemia and borderline hypoalbuminemia.

#### Xeroderma pigmentosum

Xeroderma pigmentosum (XP) is a clinical syndrome with multiple complementation groups and genotypes [72], inherited as an autosomal recessive trait [73]. The prevalence in the United States is approximately 1:250,000, and higher frequency is estimated in Japan and the Mediterranean areas [74]. XP is characterized by a variable neurological syndrome (including ataxia, choreoathetosis, spasticity, deafness, and progressive mental retardation), skin photosensitivity, early onset skin cancers, telangectasia, photophobia, conjunctivitis, keratitis, ectropion and entropion. It is typical to observe defective DNA repair after ultraviolet damage of culture cells.

The disease is genetically heterogeneous and has been classified into seven complementation groups, XPA-XPG, and each complementation group has a specific entry in the MIM database. Eight genes have been identified among XP patients [72]: seven, *XPA-XPG*, are involved in nucleotide excision repair (NER) and one, the XP variant, is involved in replication of damaged DNA of the leading strand [75].

### 4. Degenerative and progressive ARCA

In the last ten years causative genes and pathogenic mechanisms have been described for a number of degenerative and progressive ARCA. This is particularly relevant for Friedreich ataxia, the most common inherited ataxia in the Caucasian population. The defective protein in Friedreich ataxia, frataxin, is a small protein of the mitochondrial matrix for which several functions in the mitochondria have been proposed. However, there are another ataxic syndromes caused by defective mitochondrial proteins encoded by the nuclear genome. These include the X-linked sideroblastic anemia with ataxia caused by mutations in the *ABC7 *gene, the infantile onset spinocerebellar ataxia (IOSCA), and the recently described mitochondrial recessive ataxia syndrome (MIRAS).

#### Friedreich ataxia

Friedreich ataxia (FRDA) has been first described by the German pathologist Nicolaus Friedreich in nine members, two females and seven males of three sibships, in a series of five papers between 1863 and 1877 [4]. The author observed onset around puberty and presence of ataxia, dysarthria, sensory loss, muscle weakness, scoliosis, foot deformity and cardiac symptoms. After description of tendon reflexes by Erb in 1875, Friedreich also recognized the absence of these reflexes as a feature of the disease. For a number of decades, FRDA and the other forms of inherited ataxias have undergone a large debate about their clinical definition and classification. Geoffroy *et al. *in 1976 [76] and Harding in 1981 [77] proposed restricted clinical criteria for FRDA diagnosis. Harding defined essential diagnostic criteria including age of onset of symptoms before 25 years, progressive ataxia of limbs and of gait, absent knee and ankle reflexes, extensor plantar responses, dysarthria, and motor nerve conduction velocity >40 m.s^-1 ^in upper limbs with small or absent sensory action potentials. Most patients also show pyramidal weakness, absence of reflexes in upper limbs, distal loss of joint position and sense in lower limbs, scoliosis and abnormal electrocardiogram suggesting the presence of hypertrophic cardiomyopathy. Other signs are pes cavus, nystagmus, optic atrophy, deafness, and diabetes mellitus or glucose intolerance. Mapping the FRDA locus allowed to define new clinical variants that involved the main clinical criteria. The isolation of the *FRDA *gene and description of the GAA trinucleotide expansion as the main mutation have allowed to define the clinical picture and the phenotypic variability based on biological markers [78-80] (Table 2).

**Table 2:** Frequency of the clinical signs in Friedreich ataxia

**Clinical sign**	Harding^(a) ^[77] (115 patients)	Dürr *et al*.^(b) ^[78] (140 patients)	Palau^(c) ^[80] (231 patients)
	%		

Gait ataxia	100	100	100
Limb ataxia	100	99	99
Dysarthria	97	91	91
Lower limb areflexia	99	87	86
Loss of vibration sense	73	78	85
Extensor plantar response	89	79	84
Muscle weakness in lower limbs	88	67	-
Scoliosis	79	60	79
Pes cavus	55	55	77
Horizontal nystagmus	20	40	54
Saccadic-pursuit eye movements	12	30	-
Swallowing difficulties	-	27	-
Sphincter disturbances	-	23	-
Reduced visual acuity	18	13	-
Hearing loss	8	13	2
Sensitive axonal neuropathy	96	98	98
Cardiomyopathy	70 (ECG)	63 (ECG)	74 (ECG)
Abnormal brain-stem evoked potentials	-	61	-
Abnormal visual evoked potentials	-	34	-
Diabetes mellitus or glucose intolerance	10	32	13

(a) The series is based on clinical diagnosis.

(b) and (c) Data are based on genetic diagnosis (patients homozigotes for the GAA expansion)

##### Genetics and the FRDA gene

Friedreich ataxia is inherited as an autosomal recessive trait. Originally, only one locus has been recognized and mapped to chromosome 9q13 [81]. However, a rare second locus, *FRDA2*, has been proposed for some families not linked to chromosome 9 [82-84]. The *FRDA *gene spans 80 kb of genomic DNA and is composed by seven exons. A major 1.3 kb transcript is encoded by exons 1-5a and translated in a 210 amino acids protein, called frataxin [85]. A second putative transcript uses exon 5b instead of 5a followed by exon 6, but no function has yet been described.

Human mRNA frataxin is mainly expressed in spinal cord and heart, but a signal on Northern blots is also found in liver, skeletal muscle and pancreas [85]. These data correlate with the clinical presentation of the disease. Expression studies in mice (embryo and adult) confirmed the relation between the main affected tissues and frataxin expression. In embryos, expression starts in the neuroepithelium at embryonic day E10.5. Dorsal root ganglia, where the bipolar sensory cell bodies are located, are the major expression site in the nervous system, from day E14.5 to adult life. In developing mice, frataxin is also expressed in spinal cord, periventricular zone of the brain at the level of diencephalon, including cerebral cortex and the ganglionic eminence, and of the posterior mesencephalon. Non-neurological expression involves energy-dependent tissues such heart, liver, pancreas and brown adipose tissue that contain high density of mitochondria [86].

##### Molecular pathology

The most frequent mutation observed in FRDA patients is the expansion of a GAA trinucleotide repeat. The GAA tract is located within an *Alu *element belonging to the *Sx *subfamily in the first intron of the gene. Ninety six percent of patients are homozygous for GAA repeat expansions, and the remaining 4% are compound heterozygous for an expanded allele and a point mutation within the coding sequence of the gene [84,86]. Size of the mutated GAA expansion is variable between 67 to 1700 repeats.

The expanded GAA repeat results in inhibition of the *FRDA *gene expression. Reduced levels of both mRNA [85] and protein [88] have been demonstrated in tissue samples obtained from Friedreich's ataxia patients. The reduction is inversely related to the size of the GAA repeat alleles, especially to the smaller one. Long uninterrupted GAA tracts adopt triple helical structures, which can form bimolecular complexes or 'sticky' DNA formed by the association of two purine-purine-pyramidine structures [89]. Triplexes may inhibit transcription, thus providing a mechanism to explain reduced gene expression in patients [90,91].

Point mutations predicting a truncated frataxin and missense mutations have been reported [85,92-99]. Most missense mutations involved the C-terminal half of frataxin (a region better preserved in the phylogeny), suggesting that it is an important functional domain [100,101]. A number of missense mutations, L106S, D122Y, G130V, R165P, R165C and L182F, have been associated with milder and atypical clinical pictures in heterozygous patients. In most of them, the amino acid substitution is located before or after the highly conserved domain of the C-terminal frataxin. In these cases, it is likely that the less severe phenotype may be caused by a partially functional frataxin encoded by the nonexpanded allele. However, some of these amino acid changes may affect a relevant residue in the function of frataxin: one substitution involved G130 (a residue also preserved in most of the analysed species); D122 is one of the surface sites of the acidic patch, which may be important in a putative protein-protein interaction of frataxin. No mutations have been reported to involve amino acids of the peptide signal sequence, except for the first methionine [96,97], something that may be related to the absence of specific function of the N-terminal half of frataxin. Mutations in the start translation amino acid might reduce the protein production. A summary of the possible effects of missense point mutations on frataxin function (based on protein structure studies) has been reported elsewhere [102].

##### Clinical variability and molecular diagnosis

The phenotype of Friedreich ataxia does not show the homogeneity observed for other recessive traits. A number of phenotypes showing a variation in some essential clinical criteria of the classic FRDA have been mapped to the same locus on chromosome 9. These include the late onset Friedreich ataxia (LOFA) defined by onset after 25 years [103,104], FRDA with retained reflexes (FARR) [105], and the Acadian form of FRDA [106] that is characterized by slower progression rate, with no cardiomyopathy and diabetes mellitus. Analysis of the GAA repeat in these patients has confirmed FRDA diagnosis. Other unexpected presentations of FRDA (confirmed by molecular diagnosis) are pure sensory ataxia [107], spastic paraplegia [108,109] and chorea [110]. Altogether, diagnostic criteria by Geoffrey *et al. *[76] and Harding [77] remain highly specific for diagnosis of FRDA, although a few cases may be underdiagnosed when molecular diagnosis is not performed, especially those with a very late onset [111].

A correlation between the size of the GAA expansion, and the presence and timing of various features of the disease has been established. The most evident is the inverse correlation between the age of onset and the size of the smaller allele [78,79,87,112]. This correlation is not only observed with the size of the expanded allele but also with the amount of residual frataxin in lymphocytes from patients [88].

The high frequency (98%) of the GAA expansion in FRDA chromosomes makes its analysis a very helpful tool in the diagnosis of classic and variant FRDA, and in other early onset spinocerebellar ataxias. Finding two expanded GAA alleles confirms the diagnosis FRDA, whatever the phenotype, whereas a heterozygous genotype of one expanded and one non-expanded alleles is highly suggestive of FRDA. In this case, search for a point mutation is mandatory to confirm the diagnosis.

Genetic counseling is supported by the molecular diagnosis. Before the identification of the *FRDA *gene, prenatal diagnosis was based on segregation analysis of flanking linked markers in the fetal DNA of mutations found in the proband, which required DNA from both parents [113]. To date, prenatal diagnosis is feasible by direct analysis of the GAA repeat, and in very rare cases, the GAA repeat and point mutations [114]. Assuming a carrier rate in the Caucasian general population of 1:100 (2% of mutations being point mutations), prior risk for a patient to have an affected child is 1:200. By contrast, the risk is 1:10,000 if the GAA repeat is excluded in the partner. For carrier relatives, the risk to have an affected child if the partner has not a GAA expansion is 1:20,000.

##### Mitochondria and pathogenesis

Frataxin is a soluble protein with no previously known function. No specific domains related to protein families are represented in its polypeptide sequence. The protein is localized in the mitochondrial internal membrane [88], where it is processed by the mitochondrial processing peptidase (MPP) to produce the mature form with 18 kDa in a two-step process [115,116], and in the mitochondrial matrix.

Experiments in cell systems, especially in the yeast *Saccharomyces cerevisiae*, have provided relevant information about the possible function of frataxin in mitochondria and its role in the pathogenesis of the disease. At least five hypotheses for the primary mitochondrial function of frataxin have been proposed (see [117] for review): iron transport [118-120], iron-sulfur clusters (ISC) biosynthesis [121,122], iron storage [123,124], antioxidant [125,126] and stimulator of oxidative phosphorylation [127]. Some of them are discussed bellow.

The first biochemical data coming from experiments in *S. cerevisae *yeast suggested a role of the frataxin homologue gene, *YFH1*, and its encoded protein Yfh1p, in the general regulation of iron homeostasis. Knock-out yeast strains, ***Δ****YFH1*, accumulate iron in mitochondria at the expense of cytosolic iron, leading to lost of ability to carry out oxidative phosphorylation. It is thought that decreased respiratory activity and mitochondrial damage are the consequence of iron-induced oxygen radicals generated by the Fenton reaction (Fe^2+^-catalyzed production of hydroxyl radicals). Data from human studies suggested that yeast model may reflect the Friedreich ataxia pathophysiology since there is an accumulation of iron deposits in myofibrils of the heart from patients' autopsies [128], and increased iron levels in mitochondria of patients' fibroblasts [129,130]. Moreover, a reduced activity of both mitochondrial and cytosolic aconitase and complexes I, II and III have been observed in heart biopsy of two patients [121] and autopsy material of nine patients [122]. These enzymes and complexes contain iron-sulfur (Fe-S) clusters, ISC, in their active sites. Proteins containing ISC are sensitive to free oxygen radicals and may be affected by accumulation of mitochondrial iron in frataxin deficiency states.

In contrast, data from frataxin knock-out mouse models has raised some questions about the role of iron in the disease pathogenesis. Complete absence of frataxin in mouse leads to early embryonic lethality. Interestingly, no iron deposits are detected when embryos are stained with Perls technique [131]. Two conditional knock-outs in specific mouse tissues have also been reported [132]. The authors induced both striated muscle-restricted (MCK mutant) and neuron-restricted (NSE mutant) transgenic animals harbouring a frataxin exon 4 deletion. Both mutant strains together reproduce many aspects of the pathophysiology of the disease, such as cardiac hypertrophy without skeletal muscle involvement and large sensory neuron dysfunction. Deficient activities of complexes I-III of the respiratory chain and the aconitases have also been observed. However, intramitochondrial iron accumulation is time-dependent in the MCK mutant: whereas deficit of Fe-S enzymes is detected in the 7-weeks mutant mouse, mitochondrial deposit of iron is not observed until 10 weeks of age, suggesting that mitochondrial iron accumulation does not represent the primary causative pathogenic mechanism in frataxin deficiencies.

Frataxin is believed to have a function in the biogenesis of ISC. The above mentioned studies suggest that iron accumulation is a distal consequence of an earlier, proximal consequence of frataxin deficiency. It has been now clearly established that the mitochondrial iron accumulation is a general consequence of deficiencies in ISC biogenesis in yeast models [133]. Recently, deficiency of the yeast frataxin homolog protein yfh1p has been demonstrated to cause a partial defect in the maturation of mitochondrial ISC [134]. Two recent reports have demonstrated *in vitro *and *in vivo *interactions between yeast frataxin and Isu1p, the ISC scaffold protein, suggesting a main role of frataxin in the biogenesis of mitochondrial ISC [135,136]. Expression experiments using microarray technology on frataxin-deficient human cells suggest that frataxin may have a role in the ISC biogenesis and sulphur amino acid metabolism [137]. Direct or indirect role of frataxin in the ISC biogenesis may account for the biochemical phenotype observed in patient endomyocardial biopsies [121], or ROS generation. Recent studies showed that yeast frataxin increases the iron bioavailability for heme synthesis [138] and also plays a role in aconitase chaperoning [139].

Another hypothesis (based on experiments in mammalian adipocyte cells that overexpressed frataxin) suggested frataxin as an activator of mitochondrial energy conversion and oxidative phosphorylation [127]. The authors have observed a Ca^2+^-induced up-regulation of tricarboxylic acid cycle flux and respiration, and an increase in the cellular ATP content. They postulated ATP deficiency as a primary defect rather than an alteration in the mitochondrial iron homeostasis, which would be a secondary associated phenomenon. This pathogenic mechanism is in agreement with the finding of increased ATP production in postexercise skeletal muscle from FRDA patients [140]. Further experiments showing direct participation of frataxin in the respiratory chain (by interaction with protein of complex II) suggested FRDA as an oxidative phosphorylation (OXPHOS) disease [141,142].

A common point in the cell damage and death in Friedreich ataxia is thought to be the generation of free radicals and ROS. However, it has been shown that the deficiency of frataxin in a new mouse knock-out model of Friedreich ataxia does not cause oxidative stress [143]. Thus, the role of oxidative stress in the pathogenesis of the disease remains to be elucidated; moreover, the use of antioxidants is based on this hypothesis.

##### Therapeutic advances

Understanding the underlying pathogenetical mechanisms of the disease is needed to design new pharmacological therapeutic approaches. A number of drugs trying to reduce the effects of the oxidative stress (caused by intracelullar iron imbalance due to frataxin deficiency) have been introduced in the clicical practice. Both iron chelators (desferrioxamine) and antioxidants (ascorbic acid or coenzyme Q_10 _analogues) have been proposed to reduce the mitochondrial iron overload. Desferroxiamine has been used in patients with general iron overload. However, Friedriech's ataxia patients have normal plasma levels of iron and ferritin [144], although the plasma level of the transferrin receptor is increased [145]. Moreover, desferroxiamine is effective in chelating iron in the extracellular fluid and cytosol, but not in mitochondria. Thus, the usage of this chelator in FRDA remains limited and needs to be further defined.

Rustin *et al. *[146] studied *in vitro *the effect of desferrioxamine, ascorbic acid and idebenone on heart homogenates from three Friedreich's ataxia patients with valvular stenosis. Respiratory-chain complex II activity, lipoperoxidation, and mitochondrial and cytosolic aconitase activities have been tested in the presence of reduced iron (Fe^2+^), oxidazed iron (Fe^3+^), desferrioxamine, ascorbic acid, and idebenone. The authors observed decreased activity of complex II and increased lipoperoxidation in the presence of Fe^2+ ^but not Fe^3+^. Presence of idebenone protected complex II activity against iron-induced injury in membrane lipids. However, reduction of Fe^3+ ^by ascorbic acid produced peroxidation of lipids. In the same experimental system, desferrioxamine protected complex II activity from iron injury. In contrast, Fe^2+ ^decreased the aconitase activity when the chelator was present. In these experiment protocols, only idebenone (a short-chain quinone), protected the heart homogenates from iron-induced injury. Consequently, authors investigated the effect of idebenone in three patients that received idebenone (5 mg/kg daily, in three doses) for a period of 4–9 months and a reduction of the mass index in the left heart ventricle was documented by echocardiography. Further studies confirmed the improvement of hypertrophic cardiomyopathy by idebenone [147]. Lodi and colleagues [148] have treated patients with coenzyme Q_10 _and vitamin E, and have observed *in vivo *improvement of cardiac and skeletal muscle bioenergetics. Idebenone seems to have beneficial effect in Friedreich ataxia patients with cardiomyopathy but conclusive results are still not available. No significant improvement of the neurological symptoms has been observed. Longitudinal studies in this respect are currently in progress.

Schulz and colleagues [149] have detected a 2.6-fold increase of urinary 8-hydroxy-2'-deoxyguanosine (8OH2'dG) in 33 FRDA subjects, suggesting that oxidative DNA damage is also increased in these patients and that the generation of ROS may contribute to the pathogenesis of the disease. Eight of these 33 patients were treated orally with idebenone 5 mg/kg/day for eight weeks. Although no significant clinical improvement has been observed, a significant reduction to normal urinary concentration of 8OH2'dG have been reported.

Other drug therapies recently postulated include recombinant human erythropoietin (EPO), which has a broad neuroprotective and cardioprotective capacity. The use of EPO in the treatment of Friedreich ataxia is based on the increased *in vitro *frataxin expression in response to EPO [150].

#### Mitochondrial recessive ataxia syndrome

Mitochondrial recessive ataxia syndrome (MIRAS) is caused by mutations in the polymerase **γ **(*POLG*) gene, considered to be the replicative polymerase for mitochondrial DNA [151]. Most patients with MIRAS have Finnish ancestry but patients from Norway, the United Kingdom and Belgium have also been reported [152-154]. All patients are homozygous for the triptophan-to-serine substitution at position 178 (W178S) associated in *cis*-position with the E1143G polymorphism. The median age of onset is 28 years (range 5–41 years). Clinical picture may be heterogeneous. The most common manifestations include progressive gait unsteadiness, dysarthria, decreased or absent deep-tendon reflexes in the lower limbs, decreased vibration or joint position sense, nystagmus and other eye-movement abnormalities. Epilepsy and neuropathy may be initial features. Some patients may show mild to moderate cognitive impairment, involuntary movements may also be present.

Mutations in *POLG *gene have been associated with a number of clinical phenotypes. First mutation in this gene was reported in patients with autosomal dominant progressive external ophtalmoplegia (PEO) [155] but mutations have also been identified in patients manifesting Alpers syndrome [156-158], SANDO (sensory ataxic neuropathy, dysarthria, and ophtalmoparesis) [159], and other allelic phenotypes [151,152,155,160,161]. In fact, there is a POLG syndrome that shows clinical allelic heterogeneity, which in some instances is manifested as an autosomal recessive ataxic syndrome (MIRAS is one of them).

#### Infantile onset spinocerebellar ataxia

Infantile onset spinocerebellar ataxia (IOSCA), described in Finland, is characterized by a very early onset ataxia (between 1 and 2 years), athetosis and reduced tendon reflexes. Other features such as ophthalmoplegia, hearing loss, and sensory neuropathy with progressive loss of myelinated fibres in sural nerves, appear later in the disease course. Some patients show reduced mental capacity. Patients are wheelchair-bound by teens. Neuroimaging studies reveal cerebellar atrophy. This rare ataxia is caused by mutations in the *C10orf2 *gene (chromosome 10q22.3-q24.1) encoding Twinkle, a mitochondrial DNA-specific helicase, and a rarer splicing variant Twinky [162]. Mutations in this gene have also been reported in individuals with autosomal dominant progressive external ophthalmoplegia [163].

#### Charlevoix-Saguenay spastic ataxia

Autosomal recessive spastic ataxia of Charlevoix-Saguenay (ARSACS) was originally described in the North-eastern regions of Québec, Canada, in families of French ancestry [164]. In this area, the incidence at birth and the carrier rate was estimated at 1/1,932 live born infants and 1/22 inhabitants, respectively [164,165]. Clinical symptoms are early onset (often at the very early age of 12 to 18 months), progressive spastic ataxia of all limbs with paraplegia, increased tendon reflexes, dysarthria, progressive distal wasting, extensor plantar responses, reduced vibratory and positional senses, weak ankle reflexes, and horizontal gaze nystagmus with poor ocular pursuit. Electrophysiological studies show axonal and demyelinating neuropathy. Atrophy of the superior cerebellar vermis is always present. Retinal hypermyelinated fibers have been observed in patients from Québec, but absent in patients from France, Tunisia and Turkey.

The causative gene sacsin, *SACS*, maps to chromosome 13q11 [166,167]. It contains an unique very large exon of 12.7 kb encoding 11.5-kb transcript [168]. Two founder mutations, a single-base deletion at position 6594 (g.6594delT) and a g.5354C>T nonsense mutation, have been reported in individuals from North-eastern Québec. Recently, new mutations have been described in Tunisian [169], Italian [170,171], Japanese [172] and Spanish [173] patients. The presence of heat-shock domains suggests a function for sacsin in chaperone-mediated protein folding.

#### Marinesco-Sjögren syndrome

Marinesco-Sjögren syndrome (MSS) is a rare autosomal recessive disorder with approximately 200 known cases worldwide [174]. Disease onset occurs in infancy. Cardinal features of MSS are cerebellar ataxia, congenital cataracts, and retarded somatic and mental development [175]. Dysarthria, nystagmus, muscle weakness and hypotonia are frequent symptoms. Areflexia is associated with a demyelinating peripheral neuropathy. Some patients show episodes of rhabdomyolysis with sustained or episodic elevation of serum creatin kinase activity. Hypergonadotropic hypogonadism is a frequently associated feature. Muscle pathology consists of myopathic changes with rimmed vacuoles. Cerebellar cortical atrophy with vacuolated or binuclear Purkinje cells is also observed. It has been suggested that MSS with myoglobinuria and congenital cataracts-facial dysmorphism-neuropathy (CCFDN) syndromes are genetically identical, as they both map to chromosome 18qter [176,177]. In contrast, a locus for classical MSS has recently been assigned to chromosome 5q31 and mutations have been identified in the *SIL1 *gene (encoding a factor involved in the proper protein folding) [178-180]. The loss of SIL1 function results in accumulation of unfolded proteins, which are harmful to the cell.

Diagnosis is based on clinical symptoms. Ophthalmologic examination should be performed to detect cataracts. MRI scan allows investigation of cerebellar atrophy particularly involving the vermis. Muscle biopsy findings are generally non-specific. Prenatal diagnosis with molecular genetic techniques can be performed in families with know mutation. Treatment is symptomatic. Cataracts often require surgical removal to preserve vision. Hormonal replacement therapy may be needed if hypogonadism is present. Physical and occupational therapy are crucial. Patients survive to old age, with varying disability.

#### Early onset cerebellar ataxia with retained tendon reflexes

Early onset cerebellar ataxia with retained tendon reflexes (EOCARR), or Harding ataxia, was originally described by Harding in 1981 [181]. More EOCARR cases have been reported later [182-184]. Chio *et al. *[185] described 40 cases diagnosed between 1940 and 1990 in a defined area of North-western Italy. EOCARR is one of the most frequent autosomal recessive ataxias (after Friedreich ataxia and A-T): the estimated point prevalence ratio is 1/100,000 population and the birth incidence rate is 1/48,000 live births. Current data suggest that it is, in fact, a heterogeneous disorder characterized by early onset cerebellar ataxia (in the first or second decade of life) with preservation of deep tendon reflexes [186,187].

A locus on chromosome 13q11-12 has been identified in a Tunisian family, in a position where ARSACS has also been mapped. Differential diagnosis should exclude ARSACS (mutation analysis of sacsin gene), and Friedreich ataxia with retained reflexes and slow progression (analysis of the GAA repeat in the *FRDA *gene).

#### Coenzyme Q_10 _deficiency with cerebellar ataxia

This is a syndrome characterized by childhood-onset ataxia and cerebellar atrophy, and markedly reduced levels of coenzyme Q_10 _in muscle biopsies [188]. Patients associate seizures, developmental delay, mental retardation and pyramidal signs. The pattern of inheritance is thought to be autosomal recessive [189,190]. Supplementation with high dose of oral coenzyme Q_10 _may improve the clinical picture [190]. Recently, a homozygous mutation in the aprataxin gene in a family with coenzyme Q_10 _deficiency and cerebellar ataxia has been reported [191], suggesting also that coenzyme Q_10 _may participate in the pathogenesis of AOA1.

#### Posterior column ataxia and retinitis pigmentosa

Posterior column ataxia and retinitis pigmentosa (PCARP) is characterized by early onset in childhood, sensory ataxia with preservation of pain and temperature, absent reflexes, ring scotoma and progressive vision loss leading to blindness [192-194]. The locus *AXPC1 *has been mapped to chromosome 1q31 [195].

## Diagnostic methods

When suspected on the basis of clinical examination (natural history of the disease, and neurological and systemic examinations), diagnosis ARCA must be confirmed by ancillary tests such as neuroimaging (MRI, scanning) and electrophysiological examination. Neuroimaging is very useful to distinguish ARCA from developmental disorders and degenerative diseases, and to define neurological structures involved in the pathological process. For metabolic disorders associated with ataxia, specific biochemical tests or determination of enzymatic activities are required. In the last years, identification of the responsible genes for some ARCA has enabled the confirmation of the clinical diagnosis by mutation analysis, which is also used for genetic counseling.

## Differential diagnosis

A global differential diagnosis for ARCA is not reported in this review. However, it is important to underline that the differential diagnosis should establish whether one ataxic syndrome is developmental, metabolic, or degenerative. In addition, Friedreich ataxia, the most prevalent inherited ataxia, especially among the early onset cerebellar ataxias, can be excluded in a number of cases since a specific molecular test (the expansion of a GAA repeat) is available.

## Genetic counseling

As ARCA are inherited as autosomal recessive traits, previous familial history is usually no reported. The only exception could take place when parents are consanguineous or originate from the same small town or region. As these disorders usually have an early onset, genetic counseling is an important clinical tool for preventing new cases, especially for young couples with affected first child. Their risk of having an affected child in further pregnancies is 25%. Prenatal diagnosis is proposed when the disease is well diagnosed and the causative mutation in the family is identified. Pre-implantation genetic diagnosis is a new diagnostic tool but it is available only in a very few services in Europe. Genetic counseling in a healthy carrier is relevant for consanguineous partners.

## Epidemiology

Friedreich ataxia is the most common inherited ataxia in Europe, the Middle East, South Asia and North Africa, with a prevalence of 2/100,000–4/100,000. The prevalence of the early onset cerebellar ataxias (including Friedreich ataxia) and congenital ataxias has been estimated to 7.2 per 100,000 inhabitants in Cantabria, Spain [196]. Ataxia-telangiectasia is the most common autosomal recessive ataxia after Friedreich ataxia (estimated prevalence 1-2.5/100,000), followed by the early onset cerebellar ataxia with retained tendon reflexes (1/100,000).

## Management including treatments

Although for most of ARCA there is no specific treatment, for some of them a specific mediaction can be proposed. Coenzyme Q_10 _deficiency associated with ataxia may be responsive to Co Q_10 _supplementation (300 to 600 mg/day) [188-190,197,198]. Ubidecarenone (Co Q_10_) has been used in some patients with good clinical and pathologic response. Abetalipoproteinemia, which is associated with severe neurodegenerative complications, belongs to the potentially treatable or preventable conditions associated with vitamin E deficiency, similarly to ataxia with vitamin deficiency (AVED) due to mutations in the ***α***-*TTP *gene [199-201]. Abetaliproteinemia treatment is based on a diet with reduced intake of fat and a supplement of oral vitamin E at massive dosage (1,000 mg/day for infants to over 5,000 mg/day for adults) compared with normal requirements [202-204]. It seems reasonable to start the treatment with vitamin E given as **α**-tocopherol acetate 50 mg/kg/day in three divided doses. Cerebrotendinous xanthomatosis is currently treated with chenodeoxycholic acid (CDCA) but others inhibitors of the HMG-CoA reductase also reduce plasma cholestanol levels [205-207]. CDCA should be administrated in dosage of 750 mg/day (15 mg/kg/day) given in three divided doses.

## Unsolved questions

For a number of ataxic syndromes, the etiology or the causative gene remains unknown. Moreover, only few disorders, as indicated in the section "Treatment", have palliative treatment. Generation of new drugs, or gene and cell therapy approaches require a better understanding of the molecular and pathophysiological mechanisms underlying each disease.
